# Biodegradable Polylactic Acid-Polyhydroxyalkanoate-Based Nanocomposites with Bio-Hydroxyapatite: Preparation and Characterization

**DOI:** 10.3390/polym15051261

**Published:** 2023-03-02

**Authors:** Preeyaporn Injorhor, Tatiya Trongsatitkul, Jatuporn Wittayakun, Chaiwat Ruksakulpiwat, Yupaporn Ruksakulpiwat

**Affiliations:** 1 School of Polymer Engineering, Institute of Engineering, Suranaree University of Technology, Nakhon Ratchasima 30000, Thailand; 2 Research Center for Biocomposite Materials for Medical Industry and Agricultural and Food Industry, Nakhon Ratchasima 30000, Thailand; 3 School of Chemistry, Institute of Science, Suranaree University of Technology, Nakhon Ratchasima 30000, Thailand

**Keywords:** polylactic acid (PLA), polyhydroxyalkanoate (PHA), nano-hydroxyapatite, mechanical properties, thermal properties, in vitro degradation

## Abstract

Biodegradable polymers play a significant role in medical applications, especially internal devices because they can be broken down and absorbed into the body without producing harmful degradation products. In this study, biodegradable polylactic acid (PLA)-polyhydroxyalkanoate (PHA)-based nanocomposites with various PHA and nano-hydroxyapatite (nHAp) contents were prepared using solution casting method. Mechanical properties, microstructure, thermal stability, thermal properties, and in vitro degradation of the PLA-PHA-based composites were investigated. PLA-20PHA/5nHAp was shown to give the desired properties so it was selected to investigate electrospinnability at different applied high voltages. PLA-20PHA/5nHAp composite shows the highest improvement of tensile strength at 36.6 ± 0.7 MPa, while PLA-20PHA/10nHAp composite shows the highest thermal stability and in vitro degradation at 7.55% of weight loss after 56 days of immersion in PBS solution. The addition of PHA in PLA-PHA-based nanocomposites improved elongation at break, compared to the composite without PHA. PLA-20PHA/5nHAp solution was successfully fabricated into fibers by electrospinning. All obtained fibers showed smooth and continuous fibers without beads with diameters of 3.7 ± 0.9, 3.5 ± 1.2, and 2.1 ± 0.7 µm at applied high voltages of 15, 20, and 25 kV, respectively.

## 1. Introduction

The circular economy (CE) has gained attention worldwide. The focus is on balance between the economy, environment, and society, especially recycling and the use of renewable technologies and materials [1]. Nowadays, composite materials are widely used in a variety of industrial sectors, resulting in a significant accumulation of plastic waste in the environment. Plastic composites require end-of-life (EOL) treatments because they cannot be easily disposed of. So, there are various study works attempting to investigate recycling and reusing techniques for plastics and their composite materials [2,3]. On the other hand, the use of biopolymer composites does not necessitate the disposal process because they can be decomposed of by themselves. Therefore, the development of materials from renewable resources is receiving attention from many researchers [4,5,6,7,8,9].

Biopolymers derived from renewable resources such as polylactic acid (PLA), polyhydroxyalkanoate (PHA), and thermoplastic starch (TPS) are a popular alternative to traditional petroleum-based plastics in several applications [10]. Since their advantageous characteristics are renewability, biocompatibility, and biodegradability, they have been mostly used for packaging [11,12].

PLA is made from agricultural raw materials such as fermented plant starch. PLA degrades environmentally in two stages. First, high molecular weight polyester chains are hydrolyzed into oligomers, and then the oligomers are degraded into water, carbon dioxide, and humus [13]. However, PLA has limitations such as its brittleness and low heat resistance [14]. Various PLA composites with different fillers have been developed to overcome these drawbacks [15].

PHA is synthesized from bacteria (both Gram-positive and Gram-negative) that have more than 75 different genera [13]. PHA has biocompatibility, renewability, and biodegradability, but their homopolymers are brittle, with a high degree of crystallinity and narrow processability window. However, the improved PHA was used in biomedical, packaging, and agricultural plastic applications [16].

Although PLA and PHA are often used in biodegradable ap plications to ease environmental problems, some weak properties such as low mechanical properties, low thermal resistance, fragility, and low processability limit their performance. Thus, they have to blend with each other or/and be incorporated with nanoparticles for composite materials to improve their properties and performance [4,11,17,18,19,20,21,22,23]. Zhang and Thomas [17] reported that PLA blended with polyhydroxybutyrate (PHB) can improve tensile strength due to the reinforcement effect of PHB small particles.

In the last decade, nanofillers were one of the choices to overcome the drawbacks of PLA and PHA. Inorganic nanofiller such as nano-hydroxyapatite (nHAp) has received attention from many researchers. Nejati et al. [24] have synthesized nHAp/PLA composite scaffolds for bone tissue engineering. They found that nHAp can improve the elastic modulus and compressive strength of their composites. The elastic modulus and compressive strength of composites increased up to 14.9 and 8.67 MPa while that of pure PLLA increased up to 2.40 and 1.79 MPa, respectively. Liu et al. [22] have prepared PLA/nHAp composite scaffold by phase separation method. They found that nHAp can improve the thermal decomposition temperature of the composite and its hydrophilicity.

Tissue engineering refers to the attempt to create functional human tissue from cells in a laboratory. Its ultimate goal is to be a cure by repairing or replacing tissues and organs due to disease, traumatic injury, genetic errors, etc. There are four important factors that tissue engineering relies on: 1. the right cells to do the jobs, 2. the right environment such as a scaffold to support the cells, 3. the right biomolecules (growth factors), and 4. mechanical environment to influence the development of the cells [25]. Scaffolds play an important role in the cell growth. Generally, they are biocompatible and biodegradable porous structures. The scaffold requirements are biocompatibility, biodegradability, mechanical properties, scaffold architecture, and manufacturing technology [26]. So, the characteristics of biopolymers meet the requirement for scaffold development and suitable for human use. There are many research studies which developed scaffolds and medical devices from biopolymer composites for tissue engineering and biomedical applications. Fang et al. [27] developed electrospun fiber scaffolds from polycaprolactone (PCL), PLA, and HAp for osteoblast-like cells. Kara et al. [28] developed nanofibrous composite scaffolds from fish scale/poly(3-hydroxybutyrate-co-3hydroxyvalerate) (PHBV) for bone regeneration. Senatov et al. [29] developed highly porous scaffolds from PHB and HAp for small bone defects replacement in the non-load-bearing parts. Prasad et al. [30] developed biofilms from PLA incorporated with HAp for use as internal fixation. Promnil et al. [31] developed nanofibrous scaffold from PLA and silk fibroin for meniscus tissue engineering.

The functional biomaterials that are entirely derived from natural resources were expected to study and fabricate. In our previous work [9], nHAp powder was successfully prepared from fish scales. In this study, we focused on the development of PLA-PHA-based nanocomposites filled with nHAp. Effects of PHA and nHAp contents on the mechanical properties, thermal properties, and biodegradability of the composites were studied. To study the effect of nHAp contents and PHA contents on PLA-PHA/nHAp composites, PLA-20PHA-based nanocomposite filled with 0, 2.5, 5, and 10 phr of nHAp and PLA-PHA/5nHAp-based nanocomposite filled with 5, 10, and 20 phr of PHA were prepared by solution casting method. The mechanical properties of the composites including Young’s modulus, tensile strength, and % elongation at break were investigated by tensile testing. The thermal stability and thermal properties of the composites were investigated by thermogravimetric analysis (TGA) and differential scanning calorimetry analysis (DSC). Furthermore, the biodegradability of the composites was studied using in vitro degradation, which determines biodegradability by soaking the sample in phosphate buffered solution (PBS). In addition, the composites that gave the desired properties was selected to investigate electrospinnability at different applied high voltages.

## 2. Materials and Methods

### 2.1. Preparation of PLA-PHA/nHAp Composite Films

Nano-hydroxyapatite (nHAp) powder from fish scales with crystallite size of 19.41 nm was prepared in-house, as described in [9]. Briefly, fish scales were deproteinized by hydrochloric acid and treated by alkali heat treatment. Polylactic acid (PLA, Ingeo™ Biopolymer 4043D, General Purpose Grade) was supplied by NatureWorks LLC (Minnetonka, MN, USA). Polyhydroxyalkanoate (PHA, Commercial Grade) was purchased from Cuckoo Trading Hebei Co., Ltd. (Shijiazhuang, Hebei, China). Dichloromethane (DCM, Analytical grade Reagents) was purchased from Carlo Erba (Milano, Italy).

PLA-PHA/nHAp composite films were prepared by solution casting method. First, nHAp powder was dispersed in DCM using a magnetic stirrer for 24 h. PLA and PHA solutions were prepared by dissolving their pellets in DCM at a concentration of 10 wt%. Table 1 shows the formulations of PLA-PHA/nHAp composite films. Each mixture from the formulations was mixed for 72 h using a magnetic stirrer until homogenous. The PLA-PHA/nHAp solutions were poured into a Petri dish for film casting. Then, they were air-dried at room temperature for 24 h and oven-dried at 40 °C for 72 h. After that, the composite films were stored in a desiccator for further characterization and measured for their thickness. The thickness of each sample is approximately 0.50–0.70 mm.

### 2.2. Characterization of PLA-PHA/nHAp Composite Films

The mechanical properties of PLA-20PHA and PLA-PHA/nHAp composites such as tensile strength, Young’s modulus, and % elongation at break were investigated by tensile test (according to ASTM D882-10) using a universal testing machine (INSTRON/5565, Norwood, MA, USA) with a load cell of 5 kN and a crosshead speed of 250 mm/min at room temperature. Five specimens with 1 cm width and 10 cm length from each composite were performed.

The cross-section microstructure of pure PLA, PLA-20PHA, PLA/5nHAp composites, and PLA-PHA/nHAp composites were observed using a scanning electron microscope (SEM, JEOL, JSM-6010LV, Tokyo, Japan). Before testing, the cross-section of the composites after tensile testing were coated with gold sputtering.

The thermal stability of the neat PLA and PHA, PLA-20PHA, PLA/5nHAp composite, and PLA-PHA/nHAp composites was characterized using a thermal gravimetric analyzer (TGA, TGA/DSC1, Mettler Toledo, Schwerzenbach, Switzerland) under a nitrogen atmosphere from 30 °C to 500 °C with a flow rate 50 mL/min and a heating rate 10 °C/min. The TGA curves and the first derivative of TGA curves (DTG) were obtained from the analysis STAR^e^ software (version: 16.30).

Thermal properties of neat PHA, PLA-20PHA, and PLA-PHA/nHAp composites were investigated using a differential scanning calorimeter (DSC, DSC 3^+^ STAR^e^ System, Mettler Toledo, Schwerzenbach, Switzerland). The samples were heated from −50 to 200 °C with a heating rate of 10 °C/min, under nitrogen at flow rate of 50 mL/min followed by a cooling process down to −50 °C and second heating with the same procedure. The DSC thermograms provide the thermal properties such as enthalpy of melting (Δ*H*m), enthalpy of crystallization and cold crystallization (Δ*H*c, Δ*H*cc), glass transition temperature (*T*g), crystallization and cold crystallization temperature (*T*c, *T*cc), and melting temperature (*T*m). The degree of crystallinity was calculated according to Equation (1) [18]:

*Χ*c (%) = [(Δ*H*m − Δ*H*c)/(Δ*H*m^0^ × *w*)] ×100 (1)

where Δ*H*m^0^ is the heat of melting of purely crystalline PLA (93 J·g^–1^) [32] and PHA (146 J·g^–1^) [33], and *w* is the weight fraction of PLA in the sample.

In vitro hydrolytic degradation of neat PLA, PLA-20PHA, PLA/5nHAp composite, and PLA-PHA/nHAp composites was determined by soaking in phosphate buffered solution (PBS) at a concentration of 0.1 M and pH 7.4. PBS solution was prepared by dissolving 8.58 g of PBS powder (PBS powder, HiMedia, Maharashtra, India) in 1000 mL distilled water, sterilized by an autoclave at a pressure of 15 lbs at 121 °C for 15 min. The soaked specimens (10 × 10 mm) were incubated at 37 °C for 0 to 56 days. The PBS solution in all test tubes was weekly replaced by fresh PBS. The specimens were removed from PBS and wiped with a filter paper to remove surface water. Then, these specimens were rinsed by distilled water for 3 times and oven-dried at a temperature of 40 °C to a constant weight (*W*_d_). The percentage of weight loss of the specimen during immersion in PBS solution was calculated by Equation (2) [34]:

Weight loss (%) = [(*W*_0_ − *W*_d_)/*W*_0_] × 100 (2)

where *W*_0_ is an initial weight of the specimen and *W*_d_ is the weight of the specimen after removing from PBS and oven-dried at 40 °C.

### 2.3. Preparation of PLA-20PHA/5nHAp Fibers by Electrospinning Technique and Their Electrospinnability at Various Applied High Voltages

To determine electrospinnability, PLA-20PHA/5nHAp solution at a concentration of 15 wt% was fabricated to be PLA-20PHA/5nHAp electrospun fibers with an electrospinning machine (Nanon, MECC, Fukuoka, Japan). Nanofibers were spun at 90 mm distance to a drum collector, which was covered with aluminum foil. The collector rotation speed was set at 200 rpm. The high voltage between the needle tip and the drum collector was set to 15, 20, and 25 kV. The PLA-20PHA/5nHAp solution was fed at a constant flow rate of 1.0 mL/h. The electrospinning fabrication was carried out until the thickness was sufficient to be measured via diameter. The morphology of electrospun fibers were observed by SEM (JSM-6010LV, JOEL, Akishima, Tokyo, Japan). The fiber diameter was measured from SEM images using image analysis software (Image J 1.53k, Wayne Rasband and contributors, National Institutes of Health, Bethesda, MD, USA).

## 3. Results and Discussion

### Characterization of PLA-PHA/nHAp Composites

**Figure 1:**
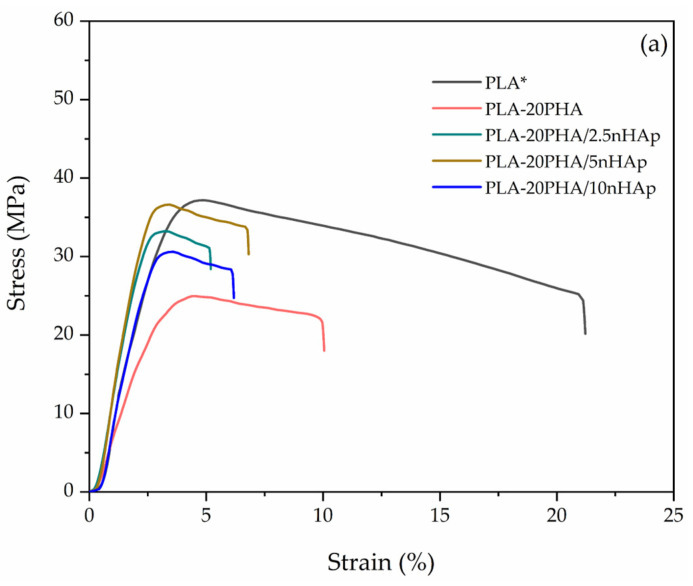

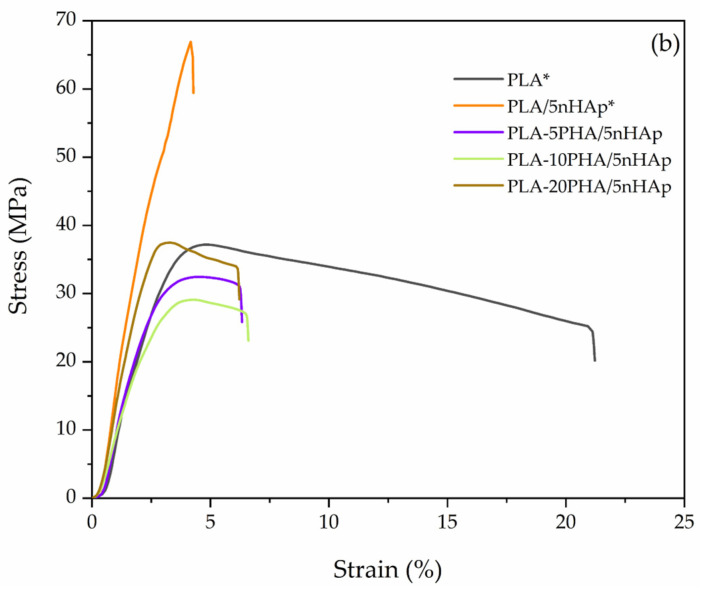
Tensile stress–strain curve of (**a**) PLA-20PHA and PLA-20PHA/nHAp composites with nHAp at various contents; (**b**) PLA/5nHAp composite and PLA-PHA/5nHAp composites with PHA at various contents. * The data were obtained from [9].

Stress-strain curves of neat PLA, PLA-20PHA, and PLA-20PHA/nHAp at various nHAp contents are shown in Figure 1a. All samples show a plastic deformation region that is attributed to a ductile fracture behavior. The mechanical properties of neat PLA, PLA-20PHA, and PLA-20PHA/nHAp at various nHAp contents are presented in Table 2. The results of neat PLA and PLA/5nHAp were obtained from previous work [9]. An increase in nHAp up to 5 phr shows the highest tensile strength (36.6 ± 0.7 MPa) and Young’s modulus (2.1 ± 0.1 GPa) which have nearly the same elongation at break as the other PLA-PHA/nHAp composites. Since nHAp is the nanoparticle filler, its large surface area has a significant impact on the physical interaction between filler and matrix. The reinforcing mechanism of nHAp in PLA matrix has been explained in previous work [9]. So, it could be assumed that the addition of nHAp, especially at 5 phr, is the optimum content that provided a well filler dispersed in the composites, resulting in enhancing the mechanical properties of the PLA-20PHA/5nHAp composite. Figure 1b shows the stress-strain curves of the PLA/5nHAp composite and PLA-PHA/5nHAp composites filled with PHA at various contents. The curves of PLA-PHA/5nHAp composites show plastic deformation region that is attributed to a ductile fracture behavior as already mentioned while the PLA/5nHAp composite shows no plastic deformation that is attributed to a brittle fracture behavior. All of the PLA-PHA/5nHAp composites show poor Young’s modulus and tensile strength compared with PLA/5nHAp composites, as shown in Table 2. It indicated immiscibility between PLA and PHA according to previous works which were observed in the literature [13,17,18,35,36]. However, their elongation at break was enhanced by the addition of PHA. In addition, the effect of PHA contents on the Young’s modulus of PLA-PHA/5nHAp composites shows that they were improved by 5 and 20 phr of PHA compared with neat PLA. It indicated that the PHA act as a reinforcement filler showing the crystalline from PHA’s reinforcement effect [13].

**Figure 2:**
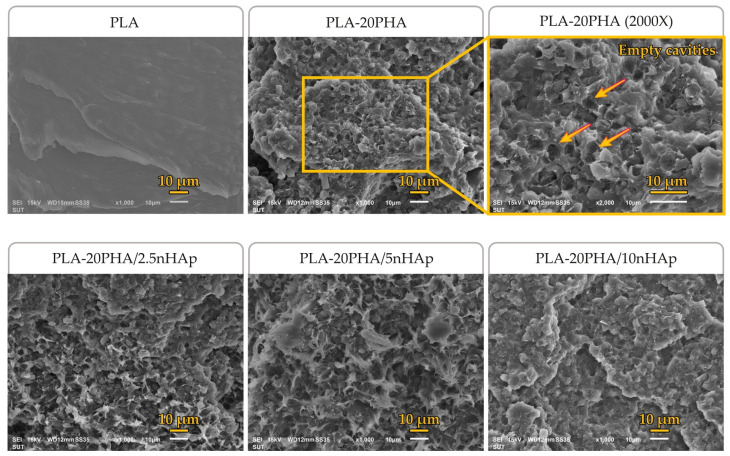
SEM micrographs of fractured surfaces of neat PLA, PLA-20PHA, and PLA-20PHA at 2000× magnification, and PLA-20PHA/nHAp with various nHAp contents.

The fractured surfaces of neat PLA, PLA-20PHA and PLA-20PHA/nHAp composites with various nHAp contents were observed by SEM. Figure 2 shows the SEM micrographs of their fracture surfaces. The neat PLA showed a smooth surface and large ligaments. On the contrary, the fractured surface of the PLA-20PHA showed roughness surface with empty cavities of spherical PHA particles which were pulled out during the fracturing [37]. Similar to the PLA-20PHA, the PLA-20PHA/nHAp composite filled with 2.5, 5, and 10 phr of nHAp also showed roughness surface with empty cavities. It could be concluded that PHA-added samples showed a lack of interfacial adhesion between PLA and PHA, inducing poor mechanical properties. This finding corresponds to tensile properties and D’Anna et al.’s report [37].

**Figure 3:**
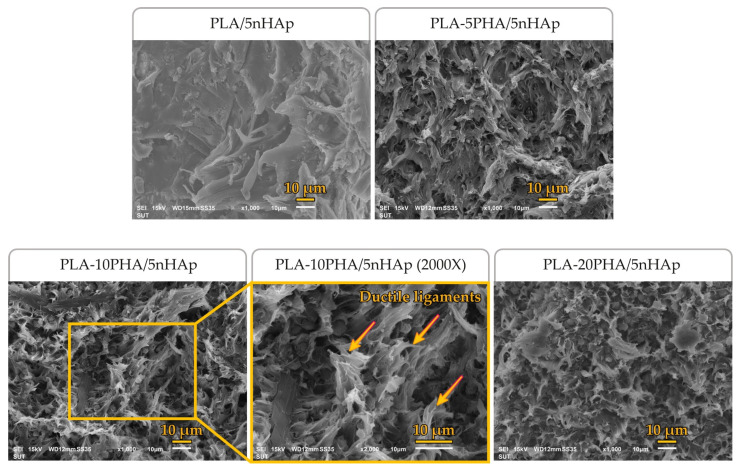
SEM micrographs of fractured surfaces of PLA/5nHAp, PLA-5PHA/5nHAp, PLA-10PHA/5nHAp, and PLA-10PHA/5nHAp at 2000× magnification, and PLA-20PHA/5nHAp.

Figure 3 shows the fractured surfaces of PLA/5nHAp composite and PLA-PHA/5nHAp composites with PHA at various contents. The presence of empty cavities and ductile ligaments was found in PHA-added samples, especially the samples with 5 and 10 PHA. These findings may be assumed that the addition of PHA induces ductile deformation resulting in the improvement of elongation at break, similar to what El-hadi [38] has reported.

**Figure 4:**
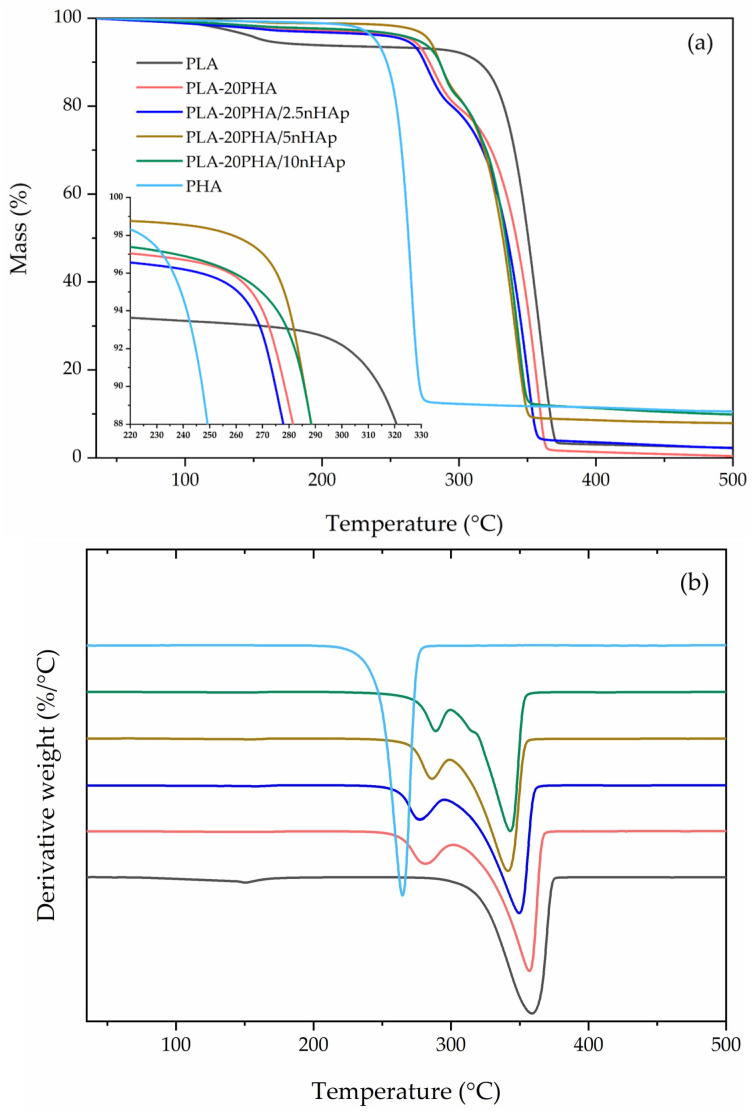
TGA (**a**) and DTG (**b**) of neat PLA and PHA, PLA-20PHA, PLA-20PHA/2.5nHAp, PLA-20PHA/5nHAp, and PLA-20PHA/10nHAp.

TGA and DTG curves of neat PLA and PHA, PLA-20PHA, and PLA-20PHA/nHAp composites with various nHAp contents are shown in Figure 4. The neat PLA and PHA show one-step degradation. In contrast, the PLA-20PHA and PLA-20PHA/nHAp with all contents of nHAp show degradation in two steps: the first one is degradation of PHA and the second one is degradation of PLA. First mass loss of the PLA-20PHA and PLA-20PHA/nHAp composites is around 17–24%. Their thermal stability was evaluated from DTG curves that are summarized in Table 3. *T*_onset_ of the PLA-20PHA/nHAp composites slightly shift to the higher temperature when nHAp content increases. Due to the extremely high thermal stability of nHAp, as shown in our previous work [9], the thermal stability of the composites in this study was improved by the high thermal stability of nHAp. According to Rakmae et al. [39], the higher thermal stability of filler acts as a barrier preventing heat transfer to the matrix. It indicated that thermal stability of the composites was improved by the addition of nHAp that has better thermal stability than PLA and PHA.

**Figure 5:**
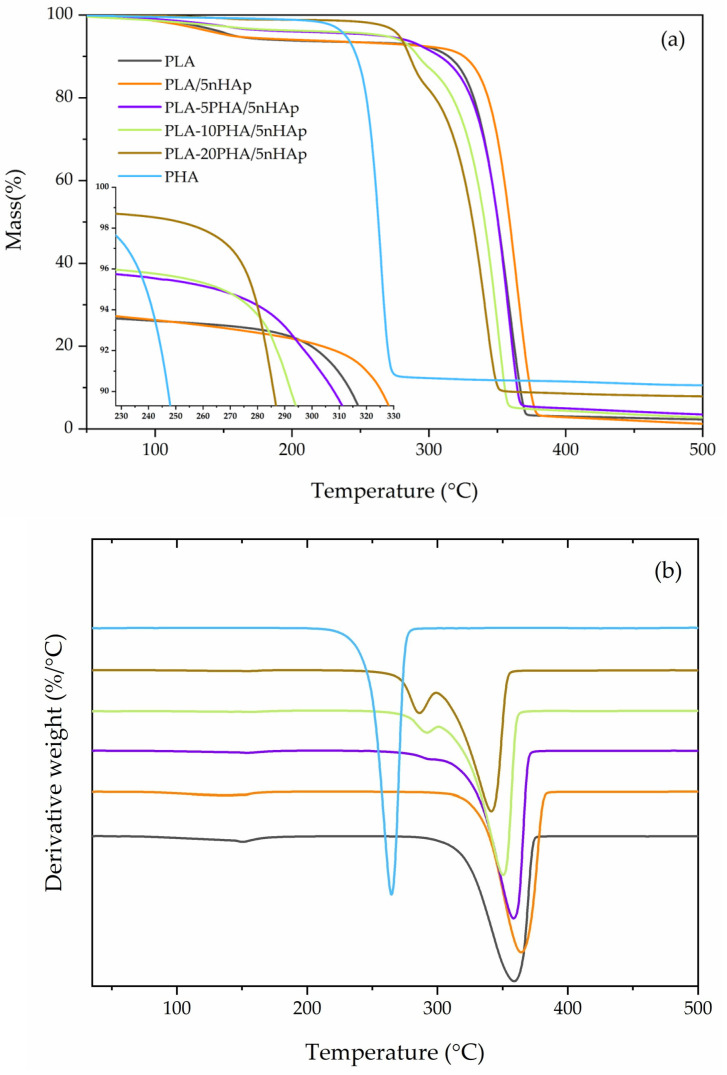
TGA (**a**) and DTG (**b**) of neat PLA and PHA, PLA/5nHAp composite, PLA-5PHA/5nHAp composite, PLA-10PHA/5nHAp composite, and PLA-20PHA/5nHAp composite.

Figure 5 shows the TGA and DTG curves of neat PLA and PHA, PLA/5nHAp composite, and PLA-PHA/5nHAp composites with various PHA contents. As shown in Table 3, PLA-PHA/5nHAp composites showed the decreasing of their *T*_onset_ when increasing PHA contents. This phenomenon was attributed to the lower thermal stability of PHA corresponding to Jimenez et al. [18]. From the results of TGA analysis, it could be concluded that the thermal stability of the composites was improved by the addition of nHAp, whereas their thermal stability was dropped by the addition of PHA.

**Figure 6:**
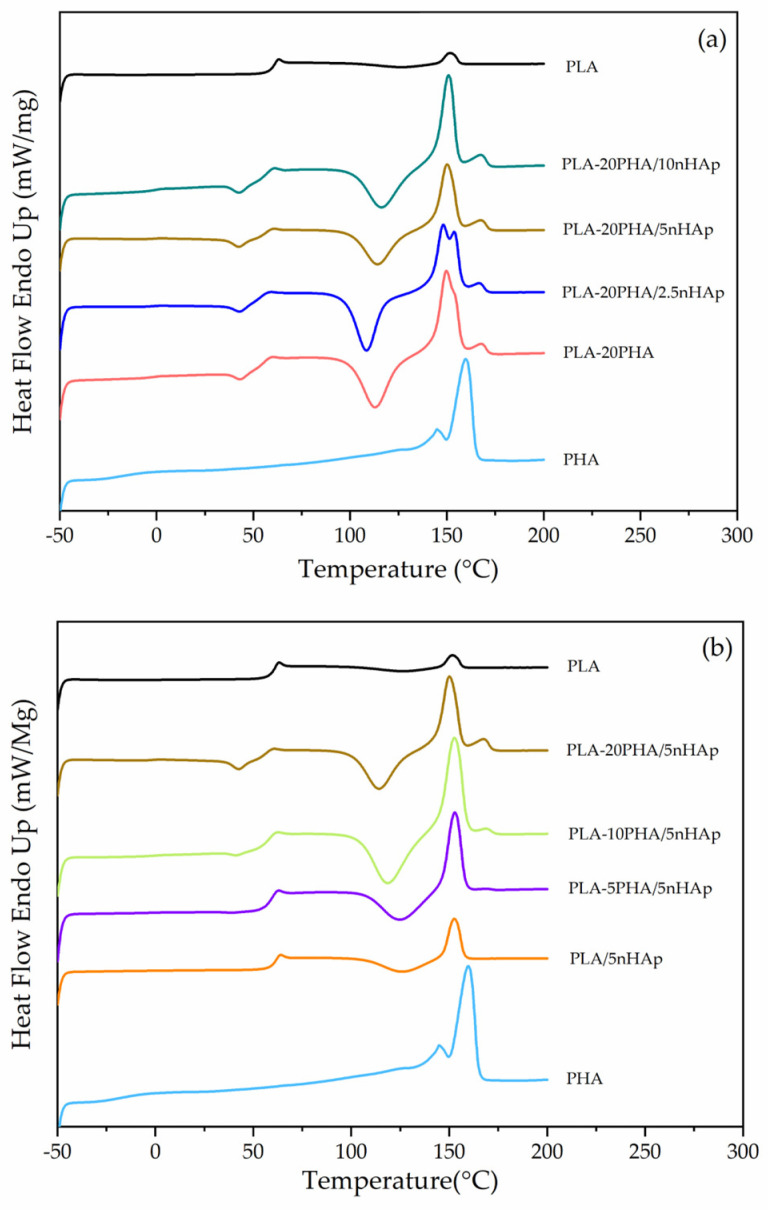
Differential scanning calorimetry (DSC) second heating curves of (**a**) neat PHA, PLA-20PHA, and PLA-20PHA/nHAp composites with various nHAp contents, and (**b**) PLA-PHA/5nHAp composites with various PHA contents.

DSC thermograms of neat PLA and PHA, PLA-20PHA, and PLA-20PHA/nHAp composites with various nHAp contents (Figure 6a), and PLA-PHA/5nHAp composites with various PHA contents are shown (Figure 6b). DSC data of all samples are summarized in Table 4. Neat PHA was observed with *T*g and *T*m at −18.00 and 159.95 °C without *T*cc, which corresponds to [40]. *T*g of PLA-20PHA/nHAp composites increased with increasing nHAp. The interfaces between organic and inorganic restricted the polymer chain motions, raising the *T*g [41]. *T*cc of PLA-20PHA/nHAp composites also increased with increasing nHAp. It suggested that nHAp particles inhibited the arrangement of the PLA-20PHA chains in a crystalline structure, resulting in a increase in *T*cc [42]. On the other hand, *T*g of PLA-PHA/5nHAp composites decreased with increasing PHA contents. It exhibited that PHA act as plasticizer in PLA-PHA/5nHAp composites, which is similar to the results of Olejnik et al. [43]. Similar to *T*g, *T*cc of PLA-PHA/5nHAp composites decreased with increasing PHA contents. In this study, the addition of PHA can promote the crystallization of the PLA-PHA/5nHAp composites, especially at 20 phr of PHA. The addition of PHA increased the crystal phase since it crystallizes as small spherulites that act as nucleating agents for PLA, causing lower *T*cc and higher crystallinity [17]. There are two visible melting peaks in the samples which were added PHA. The first peak is PLA crystal melting, while the second one is PHA crystal melting [17,43]. This phenomenon suggested that PLA and PHA are no complete miscibility [44]. However, the addition of nHAp can improve the Tm of PHA in PLA-20PHA/nHAp composites. Although the PLA and the PHA are immiscible, the thermal properties were improved by this combination.

**Figure 7:**
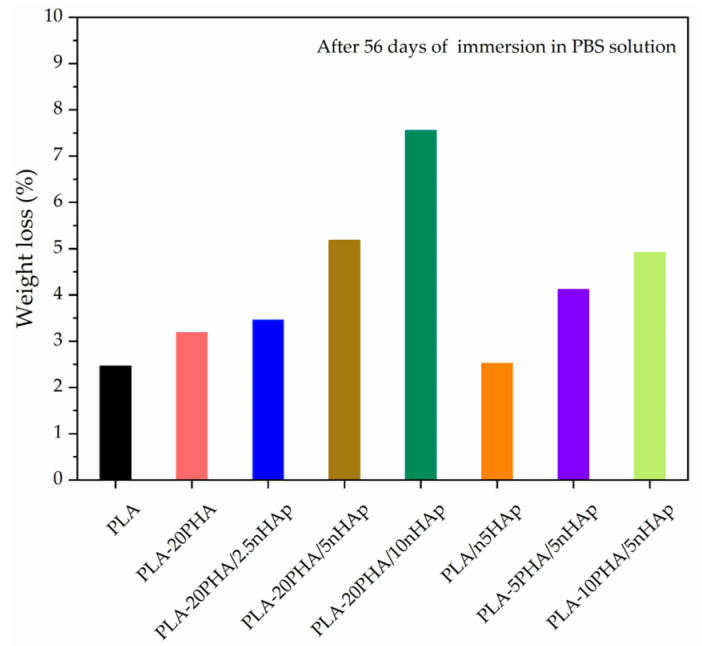
Weight loss of neat PLA, PLA-20PHA, PLA/5nHAp, and PLA-20PHA/nHAp with various nHAp contents, and PLA-PHA/5nHAp with various PHA contents after immersion in PBS solution for 56 days.

**Figure 8:**
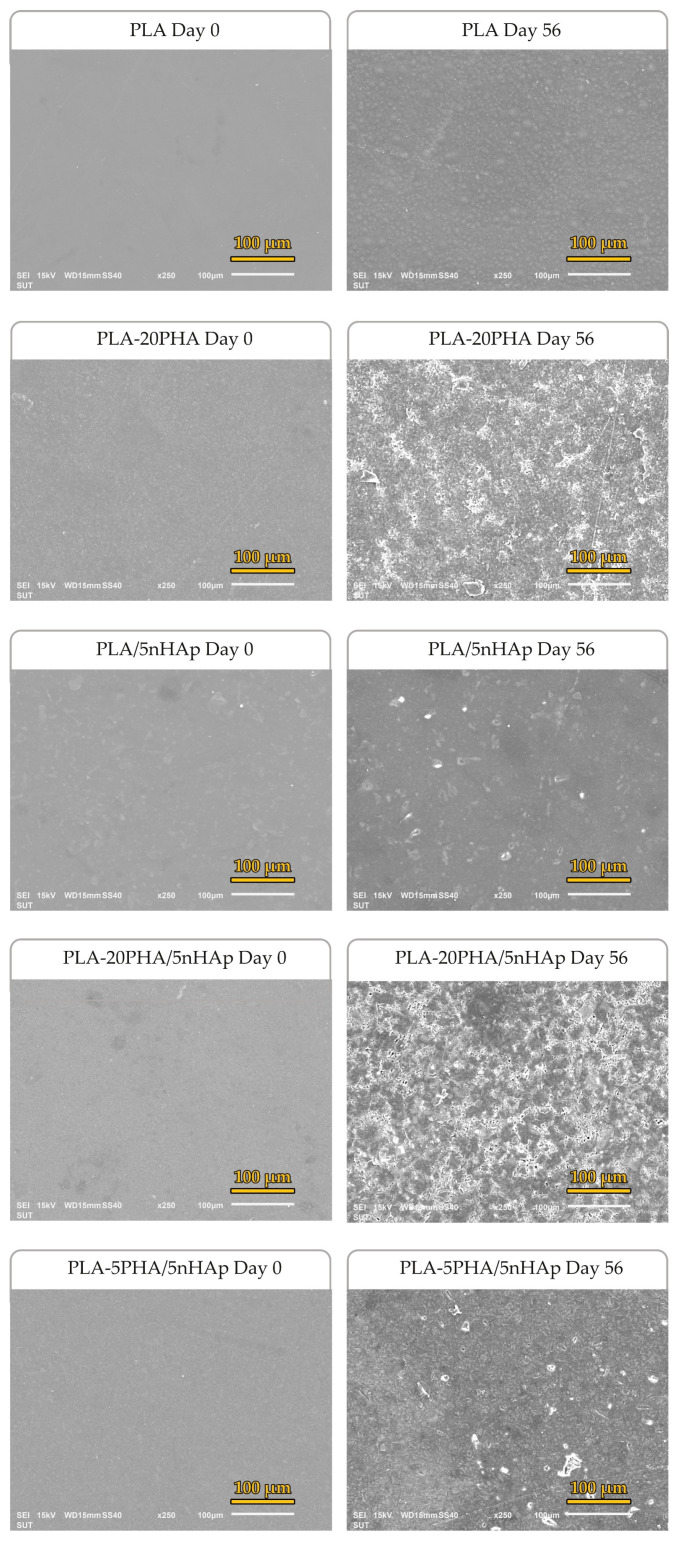
SEM micrographs of neat PLA, PLA/5nHAp, PLA-20PHA, and PLA-PHA/nHAp composite surfaces after immersion in PBS at 37 °C for 0 and 56 days.

In vitro degradation of neat PLA, PLA-20PHA, PLA-5nHAp, and PLA-PHA/nHAp composites with various nHAp and PHA contents were investigated by the percentage of weight loss of the specimens during immersion in PBS solution for 56 days as shown in Figure 7. The weight loss of neat PLA, PLA-20PHA, and PLA/5nHAp composite after 56 days is 2.46%, 3.18%, and 2.52%, respectively. The weight loss of PLA-20PHA/nHAp composite with 2.5, 5, and 10 phr of nHAp is 3.46%, 5.18%, and 7.55%, respectively. It indicated the in vitro degradation increased with increasing nHAp contents. This was due to the dissolution of nHAp and its hydrophilic properties. Moreover, the agglomeration of nHAp particles makes the liquid medium easy to access, resulting in accelerates the degradation [34,41]. The weight loss of PLA-PHA/5nHAp composites with 5, 10, and 20 phr of PHA is 4.11%, 4.91%, and 5.18%, respectively. Since higher surface area from the surface roughness is a factor influencing the biodegradation rates [16]. In this study, addition of PHA induced surface roughness of the composites. In vitro degradation increased with increasing PHA contents. However, PLA-20PHA/10nHAp showed the highest in vitro degradation. The weight loss results correspond to SEM micrographs in Figure 8. The surface of the samples have changed in morphology after immersion in PBS solution. The surface of the composites were eroded and the rough surface, crack, and hole were created, especially the composites with 20PHA. However, the blend and the composites showed more morphological change than neat PLA.

**Figure 9:**
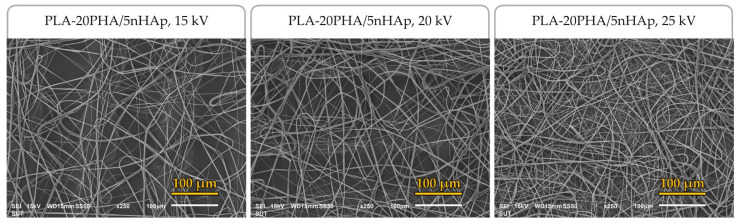
SEM images of PLA-20PHA/5nHAp composite fibers operated at different applied high voltages.

SEM images of PLA-20PHA/5nHAp composite fibers with applied high voltage at 15, 20, and 25 kV are shown in Figure 9. The continuous PLA-20PHA/5nHAp composite fibers without the formation of beads and phase separation between PLA and PHA were successfully fabricated. The average diameter of the fibers obtained from the high voltage of 15, 20, and 25 kV is 3.7 *±* 0.9, 3.5 *±* 1.2, and 2.1 *±* 0.7 µm, respectively. Since the higher applied high voltage up to the optimum value led to a decrease in the size of the Taylor cone and an increase in the jet velocity resulting in the stretching of the polymer chains, a smaller size fiber was formed [45]. It can be assumed that the fiber diameter decreased with increasing the applied high voltage as long as the applied high voltage was not adjusted over the stable stage, according to Liu et al. [46].

## 4. Conclusions

PLA-PHA-based nanocomposites filled with nHAp from fish scales were successfully prepared by the solution casting method. The dispersion of filler in the matrix and compatibility between two or more phases are significant factors in the mechanical properties of the composites. PLA-PHA-based nanocomposite with 20 phr of PHA and 5 phr of nHAp is the optimum content that gave the best mechanical performance with electrospinnability, compared to the other PLA-PHA-based nanocomposites. The addition of PHA induced thermal degradation and promoted in vitro degradation. However, the tensile strength and the thermal stability of the PLA-PHA-based composites were enhanced by the addition of nHAp. Since the composites in this study were prepared from biomaterials with medically interesting properties such as osteoconductivity from nHAp, biodegradation, and biocompatibility from PLA and PHA, they may be used as medical devices in bone tissue engineering. Additionally, PLA-20PHA/5nHAp electrospun fibers from optimal electrospinning conditions will be selected for scaffold fabrication and will be studied in vitro cell culture later.

## Figures and Tables

**Table 1:** Composite formulations with different contents of nHAp and PHA.

Designations	PLA (phr *)	PHA (phr *)	nHAp (phr *)
PLA-20PHA	100	20	−
PLA-20PHA/2.5nHAp	100	20	2.5
PLA-20PHA/5nHAp	100	20	5
PLA-20PHA/10nHAp	100	20	10
PLA-10PHA/5nHAp	100	10	5
PLA-5PHA/5nHAp	100	5	5

* phr refers to parts per hundred resins.

**Table 2:** Mechanical properties of neat PLA, PLA-20PHA, and PLA-20PHA/nHAp composites with various nHAp contents, and PLA-PHA/5nHAp composites with various PHA contents.

Designations	Young’s Modulus (GPa)	Tensile Strength (MPa)	Elongation at Break (%)
PLA *	1.7 ± 0.2	38.2 ± 1.0	23.4 ± 2.0
PLA-20PHA	1.2 ± 0.1	25.4 ± 1.2	9.9 ± 0.6
PLA-20PHA/2.5nHAp	1.9 ± 0.4	31.2 ± 1.0	5.2 ± 1.3
PLA-20PHA/5nHAp	2.1 ± 0.1	36.6 ± 0.7	5.7 ± 1.0
PLA-20PHA/10nHAp	1.7 ± 0.2	30.2 ± 2.4	5.7 ± 0.4
PLA/5nHAp *	2.7 ± 0.1	66.4 ± 3.6	4.3 ± 0.3
PLA-5PHA/5nHAp	1.8 ± 0.1	33.5 ± 1.7	6.4 ± 0.9
PLA-10PHA/5nHAp	1.3 ± 0.2	28.1 ± 1.7	6.5 ± 0.2

* The data were obtained from [9].

**Table 3:** TGA results of neat PLA and PHA, PLA-20PHA, and PLA-PHA/5nHAp composites with various PHA contents, and PLA-20PHA/nHAp composites with various nHAp contents.

Designations	*T*_onset_ (°C)	*T*_d_ PHA (°C)	*T*_d_ PLA (°C)
PLA *	330.83	−	358.83
PHA	247.59	264.67	−
PLA-20PHA	268.67	281.50	357.17
PLA-20PHA/2.5nHAp	265.33	277.33	349.67
PLA-20PHA/5nHAp	273.67	286.50	341.50
PLA-20PHA/10nHAp	275.50	289.17	343.17
PLA/5nHAp *	342.50	−	364.50
PLA-5PHA/5nHAp	332.33	293.33	358.67
PLA-10PHA/5nHAp	278.18	292.50	350.50

* The samples were obtained from [9].

**Table 4:** DSC results of neat PLA and PHA, PLA-20PHA, and PLA-20PHA/nHAp composites with various nHAp contents, and PLA-PHA/5nHAp composites with various PHA contents.

Designations	*T*g PLA (°C)	*T*c (°C)	*∆H*c (J∙g^−1^)	*T*cc (°C)	*∆H*cc (J∙g^−1^)	*∆H*m (J∙g^−1^)	*T*m PLA (°C)	*T*g PHA (°C)	*T*m1 PHA (°C)	*T*m2 PHA (°C)	*X*c PLA (%)	*X*cc PLA (%)	*X*c PHA (%)
PLA *	59.50	−	−	125.83	4.33	5.53	151.83	−	−	−	5.95	1.29	−
PHA	−	99.33	47.72	−	−	56.23	−	−18.00	144.83	159.83	−	−	5.83
PLA-20PHA	53.50	−	−	113.00	24.31	28.71	149.83	−1.85	−	168.17	37.19	5.70	−
PLA-20PHA/2.5nHAp	52.19	−	−	108.50	25.50	27.92	148.17	−2.12	153.83	167.17	36.61	3.17	−
PLA-20PHA/5nHAp	54.62	−	−	114.17	22.04	26.24	150.17	−0.81	−	167.83	35.27	5.65	−
PLA-20PHA/10nHAp	54.78	−	−	116.33	19.26	24.04	150.83	−0.73	−	168.17	33.57	6.68	−
PLA/5nHAp *	60.26	−	−	126.17	11.86	11.89	152.67	−	−	−	13.46	0.03	−
PLA-5PHA/5nHAp	57.39	−	−	124.50	16.23	16.33	152.83	−2.49	−	169.17	19.30	0.12	−
PLA-10PHA/5nHAp	56.13	−	−	118.67	22.65	24.46	152.67	−1.29	−	169.00	30.23	2.24	−

* The samples were obtained from [9].

## Data Availability

Not applicable.
